# Carcinoembryonic antigen (CEA) level, CEA ratio, and treatment outcome of rectal cancer patients receiving pre-operative chemoradiation and surgery

**DOI:** 10.1186/1748-717X-8-43

**Published:** 2013-03-01

**Authors:** Kai-Lin Yang, Shung-Haur Yang, Wen-Yih Liang, Ying-Ju Kuo, Jen-Kou Lin, Tzu-Chen Lin, Wei-Shone Chen, Jeng-Kae Jiang, Huann-Sheng Wang, Shih-Ching Chang, Lee-Shing Chu, Ling-Wei Wang

**Affiliations:** 1 Cancer Center, Taipei Veterans General Hospital, No. 201, Sec. 2, Shih-Pai Road, Taipei, 112, Taiwan; 2 Division of Colon and Rectal Surgery, Taipei Veterans General Hospital, Taipei, Taiwan; 3 Department of Pathology, Taipei Veterans General Hospital, Taipei, Taiwan; 4 Department of Nuclear Medicine, Taipei Veterans General Hospital, Taipei, Taiwan; 5 School of Medicine, National Yang-Ming University, No. 155, Sec. 2, Linong Street, Taipei, 112, Taiwan

**Keywords:** Rectal cancer, Pre-operative chemoradiation, Carcinoembryonic antigen, CEA ratio, Tumor response

## Abstract

**Background:**

To investigate serum carcinoembryonic antigen (CEA) as a prognostic factor for rectal cancer patients receiving pre-operative chemoradiotherapy (CRT).

**Methods:**

Between 2000 and 2009, 138 patients with advanced rectal cancer receiving CRT before surgery at our hospital were retrospectively classified into 3 groups: pre-CRT CEA <6 ng/ml (group L; n = 87); pre-CRT CEA ≥ 6 ng/ml and post-CRT CEA <6 ng/ml (group H-L; n = 32); and both pre- and post-CRT CEA ≥ 6 ng/ml (group H-H; n = 19). CEA ratio (defined as post-CRT CEA divided by pre-CRT CEA), post-CRT CEA level and other factors were reviewed for prediction of pathologic complete response (pCR).

**Results:**

Five-year disease-free survival (DFS) was better in groups L (69.0%) and H-L (74.5%) than in group H-H (44.9%) (p = 0.024). Pathologic complete response was observed in 19.5%, 21.9% and 5.3% of groups L, H-L and H-H respectively (p = 0.281). Multivariate analysis showed that ypN stage and pCR were independent prognostic factors for DFS and that post-CRT CEA level was independently predictive of pCR. As a whole, post-CRT CEA <2.61 ng/ml predicted pCR (sensitivity 76.0%; specificity 58.4%). For those with pre-CRT CEA ≥6 ng/ml, post-CRT CEA and CEA ratio both predicted pCR (sensitivity 87.5%, specificity 76.7%).

**Conclusions:**

In patients with pre-CRT serum CEA ≥6 ng/ml, those with “normalized” CEA levels after CRT may have similar DFS to those with “normal” (<6 ng/ml) pre-CRT values. Post-CRT CEA level is a predictor for pCR, especially in those with pre-CRT CEA ≥6 ng/ml.

## Background

Rectal cancer is one of the leading causes of cancer deaths in the world [1]. For locally advanced rectal cancer, pre-operative chemoradiotherapy (CRT) followed by radical surgery is a standard treatment [2-4]. Pre-operative CRT can improve locoregional tumor control, downstage the tumor and increase the probability of sphincter-sparing surgery [2-6]. The response of tumors to CRT varies between different patients. Tumor regression grade (TRG) is widely used to determine the tumor response to CRT in pathology [7-10]. Pathologic complete response (pCR) could be observed in 8%–25% of certain patients after regular doses of pre-operative CRT [2,3,5,6]. However, TRG and pCR can only be determined microscopically after surgery. A useful predictive model for the response of rectal cancer to pre-operative CRT has not yet been well established.

Serum carcinoembryonic antigen (CEA) is commonly measured in pre-treatment workups for rectal cancer patients [11]. The prognostic value of serum CEA levels has been widely discussed in the relevant literature: poor tumor response to CRT and an increased risk of recurrence have been observed in patients with elevated CEA levels before or after CRT [12-15]. It has also been reported that the reduction ratio of pre- to post-CRT serum CEA levels may be a prognostic factor for disease-free survival in rectal cancer patients with a pre-CRT CEA of more than 6 ng/ml [16]. However, it is rarely reported whether the clinically derived CEA parameters (including CEA reduction ratio) are correlated to pCR obtained after surgery. The purpose of this study is to evaluate the significance of serum CEA levels before treatment and their subsequent changes in predicting clinical outcomes and pathologic tumor responses for patients with rectal cancer receiving pre-operative CRT.

## Methods

### Patients

This study was approved by the institutional review board of Taipei Veterans General Hospital (No. 2011-05-0041C). Between May 2000 and July 2009, 191 patients with histologically confirmed rectal adenocarcinomas, either locally advanced disease (clinical T3, T4 or node-positive disease by AJCC staging system) or low seated primary T2 disease (<6 cm from anal verge), were treated with pre-operative CRT followed by radical surgery at Taipei Veterans General Hospital. The Eastern Cooperative Oncology Group (ECOG) performance score of all the patients was 0–2.

Among the 191 patients, 30 were excluded because of missing CEA levels after CRT; 20 were excluded due to CEA levels before or after CRT measured by enzyme immunoassay (EIA); and a further 3 receiving transanal excision instead of radical proctectomy were also excluded. The remaining 138 patients undergoing radical proctectomy with serum CEA levels measured both before and after CRT by means of radioimmunoassay (RIA) (CEA-RIACT®; CIS bio international, Gif-sur-Yvette, France), were included in this study.

Before CRT, computed tomography (CT) scans or magnetic resonance imaging (MRI, 1.5-T Siemens Vision scanner with pelvic array coil and intrarectal tube) and proctoscopy were used to evaluate the primary disease and clinical lymph node status; chest X-rays and abdominal ultrasonography were used for systemic evaluation. Among the 138 patients included in this study, pelvic CTs were done before CRT in 61 patients, and pelvic MRI s were done on the other 77 patients.

### Treatment

The detail of CRT in the protocol was described in our previous publication [6]. The prescription dose to whole pelvis was 45 Gy in 20 fractions over 4 weeks. For primary T4 disease only, a boost of 5.4 Gy in 3 fractions to the gross rectal tumors with a 1.5 cm margin followed pelvic irradiation. The median RT duration was 26 days. Oral chemotherapy agents, tegafur-uracil (UFUR; TTY Biopharm, Taipei, Taiwan) 200 mg/m^2^/day and leucovorin (Wyeth Lederle Laboratories, Taipei, Taiwan) 45 mg/day, were administered concurrently with RT (days 1–28) and after completion of RT (days 36–63; dose of tegafur-uracil adjusted to 250 mg/m^2^/day). The total daily doses of both drugs were divided into three doses per day.

At a median interval of 6.3 weeks (range, 3.4–12.4 weeks) after completion of RT, radical proctectomy with total mesorectal excision (TME) for rectal cancer was implemented. Lower anterior resection (LAR) was performed in 114 patients (82.6%) and abdominoperineal resection (APR) in 24 (17.4%), as indicated.

According to physicians’ suggestions and patients’ decisions, post-operative 5-fluorouracil based chemotherapy was implemented in 71.8% of the patients with pathologic stage III-IV and in 23.2% of the patients with pathologic stage 0-II.

### Carcinoembryonic antigen (CEA)

Serum CEA levels before CRT (pre-CRT CEA) were measured around one week before CRT, and Serum CEA levels after CRT (post-CRT CEA) were measured within one week prior to surgery. In our hospital, the normal limit of serum CEA measured by RIA was set as <6 ng/ml. According to this cutoff value, all patients were classified into 3 CEA change groups: pre-CRT CEA <6 ng/ml (group L); pre-CRT CEA ≥6 ng/ml and post-CRT CEA <6 ng/ml (group H-L); and both pre- and post-CRT CEA ≥6 ng/ml (group H-H). The extent of CEA reduction was evaluated by CEA ratio (defined as post-CRT CEA divided by pre-CRT CEA).

### Follow-up

After the completion of combined treatments, the patients were regularly followed up with physical examinations and measurement of serum CEA levels every 3–6 months for the first 2 years. Follow-up colonoscopies, pelvic CT scans, and chest radiography were also performed every 6–12 months for at least 5 years. In this study, recurrences were diagnosed either pathologically or radiologically.

### Statistical analysis

Statistical Package for Social Sciences software (SPSS version 17.0, Chicago, IL) was used. Chi-square test, Fisher’s exact test, independent *t*-test or one-way analysis of variance (ANOVA) was implemented to analyze variables. Local control (LC) and disease-free survival (DFS) from the time of surgery were calculated by Kaplan-Meier method, using log-rank tests for comparison. Univariate and multivariate analysis by Cox proportional hazards model or logistic regression were performed. Receiver operating characteristic curves (ROC), involving the Youden index (maximum [sensitivity + specificity - 1]), were used to determine optimal cutoff values. A *p*-values <0.05 (two-sided test) was considered significant.

## Results

### Overall characteristics of the patients

Of the 138 patients, 98 (71%) were males. The mean age was 63 (range 33–83 years), and mean pre- and post-CRT CEA levels (ng/mL) were 13.2 (range, 1.3–400.0) and 3.7 (range, 0.4–39.5) respectively. Free resection margin were found in all surgical specimens. Twenty-five patients (18%) achieved pCR after CRT. The median follow-up time from the start of RT was 59 months (range, 3–141 months).

### Clinicopathologic features, local control and disease-free survival of patients with reference to CEA change groups

According to pre- and post-CRT CEA levels, 138 patients in this study were retrospectively categorized into 3 CEA change groups as previously defined: group L (n = 87), group H-L (n = 32) and group H-H (n = 19). The mean pre-CRT CEA levels (ng/ml) were 3.2 (range, 1.1–6.0), 33.7 (range, 6.3–400.0) and 24.6 (range, 6.4–110.0) for group L, group H-L and group H-H respectively; the mean post-CRT CEA levels (ng/ml) were 2.6 (range, 0.4–7.3), 3.3 (range, 0.8–5.9) and 9.3 (range, 6.1–39.5) for group L, group H-L and group H-H respectively. Only 1 patient in group L had a post-CRT CEA of more than 6 ng/ml (7.3 ng/ml). The clinicopathologic features between the 3 groups are shown in Table 1, The median follow-up time from the start of RT was 60 months (range, 8–141 months), 58 months (range, 10–127 months) and 49 months (range, 3–124 months) for group L, group H-L and group H-H respectively.

**Table 1:** Clinicopathologic features between group L, group H-L and group H-H

	**Group L (n = 87)**	**Group H-L (n = 32)**	**Group H-H (n = 19)**	***p*-value**
Gender				
Male	59 (67.8%)	22 (68.8%)	17 (89.5%)	0.161
Female	28 (32.2%)	10 (31.2%)	2 (10.5%)	
Age (years), mean (range)	62 (33–83)	65 (42–81)	64 (44–78)	0.351
Clinical T stage				
cT2	15 (17.2%)	3 (9.4%)	1 (5.3%)	0.362
cT3-4	72 (82.8%)	29 (90.6%)	18 (94.7%)	
Clinical N stage				
cN0	26 (29.9%)	7 (21.9%)	1 (5.3%)	0.072
cN1-2	61 (70.1%)	25 (78.1%)	18 (94.7%)	
Clinical Stage Grouping				
Stage I-II	24 (27.6%)	7 (21.9%)	1 (5.3%)	0.111
Stage III-IV ^†^	63 (72.4%)	25 (78.1%)	18 (94.7%)	
Pathologic T stage				
ypT0, Tis, T1-2	49 (56.3%)	15 (46.9%)	6 (31.6%)	0.131
ypT3-4	38 (43.7%)	17 (53.1%)	13 (68.4%)	
Pathologic N stage				
ypN0	63 (72.4%)	28 (87.5%)	9 (47.4%)	0.008
ypN1-2	24 (27.6%)	4 (12.5%)	10 (52.6%)	
Pathologic Stage Grouping				
yp stage 0 ^*^	19 (21.8%)	7 (21.9%)	1 (5.3%)	0.030
yp stage I-II	43 (49.5%)	21 (65.5%)	8 (42.1%)	
yp stage III-IV ^§^	25 (28.7%)	4 (12.5%)	10 (52.6%)	
pCR	17 (19.5%)	7 (21.9%)	1 (5.3%)	0.281
Distance from anal verge (cm), mean (range)	6.0 (0.5–14.0)	6.5 (3.0–14.0)	6.7 (3.0–12.0)	0.388
Surgery type				
APR	17 (19.5%)	4 (12.5%)	3 (15.8%)	0.655
LAR	70 (80.5%)	28 (87.5%)	16 (84.2%)	

Abbreviations: pCR, pathologic complete response; APR, abdominoperineal resection; LAR, low anterior resection.

^†^ One patient was staged as clinical stage IV due to suspected lymph nodes at the root of inferior mesenteric artery. The patient was classified into group H-H.

^*^ Including 25 patients with ypT0N0 cM0 and another 2 patients with ypTisN0 cM0.

^§^ Three patients were proved to be pathologic stage IV. Two of them were classified into group L, and the other into group H-H.

**Figure 1:**
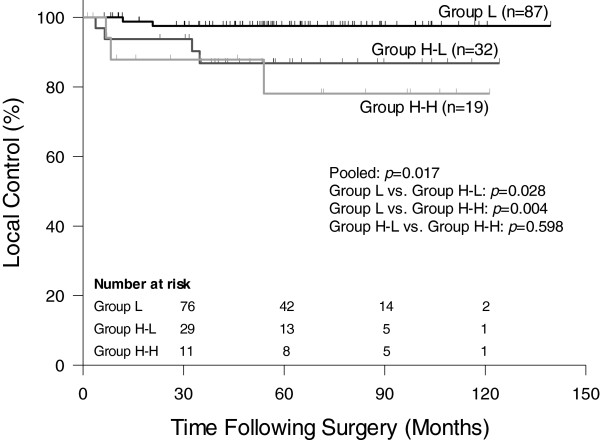
**Comparison of local control between group L, H-L and H-H.** Group L: pre-CRT CEA <6 ng/ml; Group H-L: pre-CRT CEA ≥6 ng/ml and post-CRT CEA <6 ng/ml; Group H-H: both pre- and post-CRT CEA ≥6 ng/ml.

**Figure 2:**
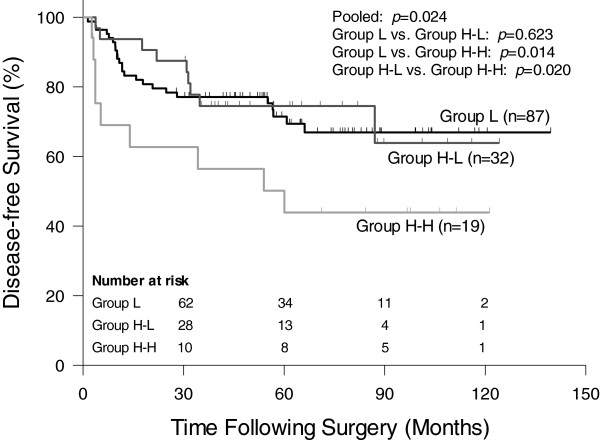
**Comparison of disease-free survival between group L, H-L and H-H.** Group L: pre-CRT CEA <6 ng/ml; Group H-L: pre-CRT CEA ≥6 ng/ml and post-CRT CEA <6 ng/ml; Group H-H: both pre- and post-CRT CEA ≥6 ng/ml.

The 5-year LC rate was 97.5% in group L, which was significantly better than the 86.8% in group H-L and the 78.1% in group H-H (*p* = 0.017, Figure 1). The 5-year DFS rate was similar in group L (69.0%) and group H-L (74.5%), but significantly lower in group H-H (44.9%) (*p* = 0.024, Figure 2). In univariate analysis, potential predictors for DFS included ypT stage, ypN stage, pCR, and CEA change groups. In multivariate analysis, only ypN stage and pCR were independently predictive of DFS (Table 2).

**Table 2:** Univariate and multivariate analysis of predictive factors for DFS (n = 138)

	**Univariate analysis**		
**Variable**	**No. of patients**	**5-year DFS (%)**	***p*value**
Gender			
Male	98	66.5	0.775
Female	40	68.2	
Age			
≤60	57	61.1	0.461
>60	81	72.0	
Clinical T stage			
cT2	19	82.0	0.089
cT3-4	119	64.5	
Clinical N stage			
cN0	34	76.0	0.235
cN1-2	104	64.0	
Pathologic T stage			
ypT0, Tis, T1-2	70	81.5	<0.001
ypT3-4	68	51.3	
Pathologic N stage			
ypN0	100	80.1	<0.001
ypN1-2	38	32.5	
pCR			
Yes	25	94.4	0.001
No	113	60.7	
Distance from anal verge			
≤6 cm	82	64.9	0.847
>6 cm	56	69.9	
Surgery type			
APR	24	57.6	0.346
LAR	114	69.8	
CEA change groups			
Group L	87	69.0	0.024
Group H-L	32	74.5	
Group H-H	19	44.9	
			**Multivariate analysis**		
**Variable**	**Hazard ratio**	**95% CI**	***p*value**	
Pathologic N stage				
ypN0	1			
ypN1-2	3.300	1.840–5.916	<0.001	
pCR				
Yes	1			
No	8.502	1.143–63.228	0.037	

Abbreviations: DFS, disease-free survival; CI, Confidence interval; pCR, pathologic complete response; CRT, chemoradiotherapy; RT, Radiotherapy; CEA, carcinoembryonic antigen.

### Clinical predictors of pCR after chemoradiation

**Figure 3:**
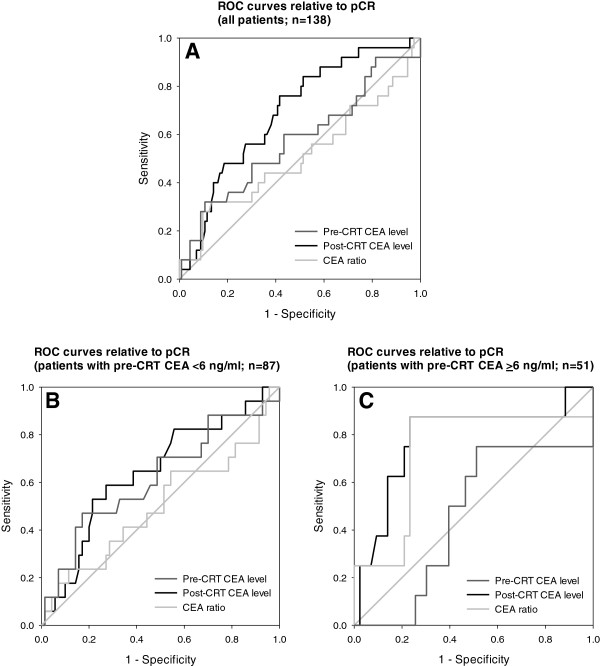
**Receiver operating characteristic curves of various CEA parameters relative to pathologic complete response.** (**A**) For all patients (n = 138), the area under the curves (AUCs) were 0.575 (*p* = 0.243), 0.691 (*p* = 0.003) and 0.508 (*p* = 0.894) for pre-CRT CEA, post-CRT CEA and CEA ratio, respectively. (**B**) For those with pre-CRT CEA <6 ng/ml (n = 87), the AUCs were 0.617 (*p* = 0.135), 0.644 (*p* = 0.066) and 0.501 (*p* = 0.991) for pre-CRT CEA, post-CRT CEA and CEA ratio, respectively. (**C**) For those with pre-CRT CEA ≥6 ng/ml (n = 51), the AUCs were 0.459 (*p* = 0.717), 0.783 (*p* = 0.012) and 0.733 (*p* = 0.038) for pre-CRT CEA, post-CRT CEA and CEA ratio, respectively.

We review possible clinical parameters that may predict pCR. In univariate logistic regression (Table 3), potential predictors for pCR included gender and post-CRT CEA level. In multivariate logistic regression, post-CRT CEA level was independently predictive of pCR, with an odds ratio 0.605 (range, 0.412–0.890; p = 0.011). When performing ROCs of various CEA parameters relative to pCR (Figure 3A), post-CRT CEA level was also the only significant predictor with area under the curve (AUC) of 0.691 (*p* = 0.003), and its optimal cutoff value was 2.61 ng/ml (sensitivity 76.0%; specificity 58.4%) with the maximum Youden index (0.344). We also observed the patients with post-CRT CEA level <2.61 ng/ml (n = 66) had better overall survival than those with post-CRT CEA level ≥2.61 ng/ml (n = 72) (5-years overall survival, 89.3% vs. 67.9%; *p* = 0.005).

**Table 3:** Univariate and multivariate analysis of predictive factors for pCR (n = 138)

	**Univariate analysis**		
**Variable**	**Odds ratio**	**95% CI**	***p*-value**
Gender			
Male	0.357	0.146–0.872	0.024
Female	1		
Age	1.016	0.979–1.054	0.403
Clinical T stage			
cT2	1.244	0.375–4.129	0.721
cT3-4	1		
Clinical N stage			
cN0-1	0.527	0.167–1.661	0.274
cN2	1		
Clinical stage grouping			
Stage I-II	0.578	0.183–1.829	0.351
Stage III-IV ^†^	1		
Distance from anal verge	0.949	0.794–1.136	0.571
RT-surgery interval	1.028	0.991–1.066	0.138
Pre-CRT CEA level	1.009	0.997–1.020	0.152
Post-CRT CEA level	0.676	0.478–0.955	0.026
CEA change groups			
Group L	4.371	0.545–35.069	0.165
Group H-L	5.040	0.569–44.636	0.146
Group H-H	1		
CEA ratio	0.923	0.290–2.938	0.892
	**Multivariate analysis**		
**Variable**	**Odds ratio**	**95% CI**	***p*-value**
Post-CRT CEA level	0.605	0.412–0.890	0.011

Abbreviations: pCR, pathologic complete response; CRT, chemoradiotherapy; CEA, carcinoembryonic antigen; CI, confidence interval; RT, radiotherapy.

^†^ One patient was staged as clinical stage IV due to suspected lymph nodes at the root of inferior mesenteric artery.

For those with pre-CRT CEA <6 ng/ml (n = 87; Figure 3B), none of the ROC curves of various CEA parameters was significantly predictive of pCR. For those with pre-CRT CEA ≥6 ng/ml (n = 51; Figure 3C), the ROC curves of post-CRT CEA level and CEA ratio were both significantly predictive of pCR with AUCs of 0.783 (*p* = 0.012) and 0.733 (*p* = 0.038), respectively.

## Discussion

Serum CEA concentrations are usually measured for rectal cancer patients. Measurement of serum CEA levels is inexpensive, standardized for normal limit, widely used and easily performed, as compared with other potential prognostic markers for rectal cancer patients, such as CA 19–9, p53, ras expression, thymidine synthase, dihydropyrimidine dehydrogenase, 18q loss of heterozygosity, deleted in colon cancer (DCC) protein, DNA ploidy or micro-RNA signature [11,17,18]. The micro-RNA signature may be of value in predicting pCR [18], but will need further validation. Several studies had focused on the predictive value of pre- and post-CRT CEA levels in patients with rectal cancer receiving pre-operative CRT [12-15,19,20]. In this study, we found not only pre-CRT CEA levels had prognostic significance, “normalization” of these values and CEA ratio also predicted tumor response and may be helpful in the design of individualized treatment for rectal cancer with high CEA levels before treatment.

There is some controversy as to the role of pre-CRT CEA in rectal cancer patients. It was reported that pre-CRT CEA levels >2.5 or >5 ng/ml were associated with poor pCR rates and poor disease-free survival on univariate analysis, but not for both on multivariate analysis [12,13]. However, in other studies, pre-CRT CEA levels were a common predictor of downstaging, pCR and tumor response on multivariate analysis [14,15]. As the pre-CRT CEA levels increase, the rates of good response might decrease. In a recent study, for pre-CRT CEA <3, 3–6, 6–9 and >9 ng/ml, the rates of good response were 36%, 24%, 16% and 8%, respectively [20]. On the other hand, post-CRT CEA level >5 ng/ml was associated with decreased pCR rates and disease-free survival on univariate and multivariate analysis [12,19]. The literature mentioned above suggests that pre- or post-CRT CEA level 3–6 ng/ml may serve as a valuable threshold for prognosis and prediction of pathologic tumor response. The change of CEA levels before and after CRT were also investigated in some Korean studies [16,20]. Park et al. categorized locally advanced rectal cancer patients into 3 groups according to pre- and post-CRT CEA levels (> or ≦3 ng/ml). They concluded that these groupings could be of clinical value as a predictor of response (TRG) to preoperative CRT and as an independent prognostic factor. However, the idea of CEA reduction ratio (like our CEA ratio) had not been previously mentioned until Kim’s study. They also classified rectal cancer patients into 3 groups according to pre- and post-CRT CEA levels, though with different cut off values (> or ≦6 ng/ml), For patients with pre-CRT CEA >6 mg/ml, they were further divided by whether post-CRT CEA was ≥70% lower than pre-CRT CEA. Similarly, they had a better 5-year DFS for the lower pre-CRT group and higher pre-CRT group with a CEA reduction ratio ≥ 70% than the other group. However, they could not definitely explain the pathological basis behind the CEA reduction ratio; and it was unknown whether pCR were related to this ratio.

Our study implied that pre-CRT CEA levels may be prognostic of local tumor control but may not be predictive of pCR. Higher pre-CRT CEA levels could be related to more advanced locoregional spread and thus associated with poorer local control, but not necessarily reflect sensitivity to CRT. The 5-year DFS rates of groups L, H-L and H-H among our patients were compatible with the two Korean studies mentioned above. Group H-H carried significantly higher risks of ypN1-2 disease and pathologic stage III-IV than groups L and H-L. This could explain why group H-H had the worst 5-year DFS rate of the three groups. Both ypN stage and pCR were parameters representing tumor response to CRT and, in our multivariate analysis, were independent prognostic factors for DFS. Though CEA change groups were not, this grouping made before surgery was obviously related to these two parameters (Table 1). Perhaps, the small number of patients may also make CEA change group statistically less prognostic. Furthermore, both ypN stage and pCR are pathologic features, limiting their prognostic value before operation.

We tried to find a clinical parameter predicting the pathologic response. CEA change groups were relevant to pCR, but may not be significant enough. Interestingly, we found male gender was a negative predictor for pCR in univariate analysis, although not anymore in multivariate analysis. Post-CRT CEA level was the only independent predictor for pCR in our data. By using ROC curves, post-CRT CEA level was a better predictor than pre-CRT CEA level or CEA ratio (Figure 3A), and the optimal cutoff value of post-CRT CEA was 2.61 ng/ml. However, when restricting to those with pre-CRT CEA <6 ng/ml, this observation was weakened and not statistically valuable (Figure 3B). On the other hand, for those with pre-CRT CEA ≥6 ng/ml, the AUC was even larger for post-CRT CEA level to predict pCR, and CEA ratio had value as good as post-CRT CEA level did (Figure 3C). This suggested that the pathologic tumor response was related to post-CRT CEA levels rather than to pre-CRT CEA levels, especially for those with high pre-CRT CEA levels.

RT-surgery interval is a well-known predictor for tumor response to pre-operative CRT, but we did not identify this even in univariate analysis, which could be due to less variance of the interval in our patients. The mean RT-surgery intervals of the patients with post-CRT CEA <2.61 ng/ml and those with post-CRT CEA ≥2.61 ng/ml were 44.6 days (range, 27–87) and 44.8 days (range, 24–79), respectively (*p* = 0.946). The comparable results supported that post-CRT CEA groups could predicted pCR independently. Besides, when dividing all patients into two groups according to the post-CRT CEA cutoff value 2.61 mentioned above, lower post-CRT CEA group correlated with earlier pathologic stages (*p* = 0.031; not shown in the result section) and better overall survival (*p* = 0.005).

The change of serum CEA levels before and after CRT seems to be more obvious in patients with pre-CRT serum CEA ≥6 ng/ml than those with normal levels (mean CEA ratio ± standard deviation, 0.36 ± 0.25 vs. 0.87 ± 0.30; *p* < 0.001); accordingly, a low post-CRT CEA level or CEA ratio may represent a marked reduction of tumor burden after CRT, especially when pre-CRT CEA levels are higher than the normal limit. We observed pCR may be even more predictable (larger AUC) when using post-CRT CEA level or CEA ratio in those with pre-CRT CEA levels ≥6 ng/ml; the optimal cutoff values of post-CRT CEA level and CEA ratio were 2.87 ng/ml and 0.22, respectively (not shown in the result section), with the same sensitivity 87.5%, specificity 76.7% and maximum Youden index (0.642). But, it was still not reasonable to conclude that surgery could be totally omitted for those with elevated pre-CRT CEA and low post-CRT CEA levels (i.e. low CEA ratios). We hypothesized that significant CEA reduction may play a role in supporting significant tumor regression for patients with both high pre-CRT CEA and clinical good response after CRT. For some highly selected cases like these, more conservative surgery may be used to preserve the sphincter without compromising local control [21].

In this study, all the patients were treated according to the same treatment protocol of pre-operative CRT, which had been proved to be effective and tolerable [6]. However, several limitations to this study exist, including retrospective design, relatively small patient numbers, imbalance of case numbers in different groups, variation in RT-surgery intervals, and inconsistent principle and regimen for adjuvant chemotherapy. Many patients were excluded due to absence of serum CEA levels or due to different laboratory techniques for measurement.

## Conclusions

In conclusion, for locally advanced rectal cancer patients with pre-treatment CEA levels ≥6 ng/ml, “normalization” of these values after CRT may predict similar tumor response and DFS to that of those patients with pre-CRT CEA <6 ng/ml. Those patients with persistent high CEA level after CRT would have the poorest response and worst DFS. We hypothesize that low post-CRT CEA levels obtained before surgery may predict pCR in rectal cancer patients receiving pre-operative CRT, while CEA ratios may also predict pCR only in patients with pre-CRT serum CEA ≥6 ng/ml.

## Abbreviations

CEA: Carcinoembryonic antigen; CRT: Chemoradiotherapy; DFS: Disease-free survival; pCR: Pathologic complete response; TRG: Tumor regression grade; AJCC: American Joint Committee on Cancer; ECOG: Eastern Cooperative Oncology Group; EIA: Enzyme immunoassay; RIA: Radioimmunoassay; CT: Computed tomography; MRI: Magnetic resonance imaging; LAR: Lower anterior resection; APR: Abdominoperineal resection; SPSS: Statistical Package for Social Sciences software; ANOVA: Analysis of variance; LC: Local control; ROC: Receiver operating characteristic curve; AUC: Area under the curve; DCC: Deleted in colon cancer; CI: Confidence interval; RT: Radiotherapy.

## Competing interests

The authors indicated no potential conflicts of interests.

## Authors’ contributions

KLY were responsible for data collection and statistic analysis, making figures and tables, and writing the manuscript. SHY, JKL, TCL, WSC, JKJ, HSW and SCC made diagnosis, staged the diseases and performed surgery to get specimens. WYL & YJK were responsible for pathological review of the tumor regression grade. LSC was responsible for the serum CEA measurement and provided the information of measurement kit. LWW had the original data and was responsible for study concept, study design, supervision and confirming the final manuscript. All authors have read and approved the manuscript.

## References

[B1] Jemal A Bray F Center MM Ferlay J Ward E Forman D Global cancer statistics CA Cancer J Clin 2011 61 69 90 10.3322/caac.20107 21296855

[B2] Sauer R Becker H Hohenberger W Rodel C Wittekind C Fietkau R Martus P Tschmelitsch J Hager E Hess CF Karstens JH Liersch T Schmidberger H Raab R Preoperative versus postoperative chemoradiotherapy for rectal cancer N Engl J Med 2004 351 1731 1740 10.1056/NEJMoa040694 15496622

[B3] Gerard JP Conroy T Bonnetain F Bouche O Chapet O Closon-Dejardin MT Untereiner M Leduc B Francois E Maurel J Seitz JF Buecher B Mackiewicz R Ducreux M Bedenne L Preoperative radiotherapy with or without concurrent fluorouracil and leucovorin in T3-4 rectal cancers: results of FFCD 9203 J Clin Oncol 2006 24 4620 4625 10.1200/JCO.2006.06.7629 17008704

[B4] Bosset JF Collette L Calais G Mineur L Maingon P Radosevic-Jelic L Daban A Bardet E Beny A Ollier JC Chemotherapy with preoperative radiotherapy in rectal cancer N Engl J Med 2006 355 1114 1123 10.1056/NEJMoa060829 16971718

[B5] Kao PS Chang SC Wang LW Lee RC Liang WY Lin TC Chen WS Jiang JK Yang SH Wang HS Lin JK The impact of preoperative chemoradiotherapy on advanced low rectal cancer J Surg Oncol 2010 102 771 777 10.1002/jso.21711 20872811

[B6] Wang LW Yang SH Lin JK Lin TC Chan WK Chen WS Wang HS Jiang JK Lee RC Li AF Chao Y Chi KH Yen SH Pre-operative chemoradiotherapy with oral tegafur-uracil and leucovorin for rectal cancer J Surg Oncol 2005 89 256 263 discussion 263–254 10.1002/jso.20168 15726610

[B7] Ryan R Gibbons D Hyland JM Treanor D White A Mulcahy HE O'Donoghue DP Moriarty M Fennelly D Sheahan K Pathological response following long-course neoadjuvant chemoradiotherapy for locally advanced rectal cancer Histopathology 2005 47 141 146 10.1111/j.1365-2559.2005.02176.x 16045774

[B8] Bouzourene H Bosman FT Seelentag W Matter M Coucke P Importance of tumor regression assessment in predicting the outcome in patients with locally advanced rectal carcinoma who are treated with preoperative radiotherapy Cancer 2002 94 1121 1130 10.1002/cncr.10327 11920483

[B9] Bozzetti F Andreola S Bertario L Pathological features of rectal cancer after preoperative radiochemotherapy Int J Colorectal Dis 1998 13 54 55 10.1007/s003840050134 9548104

[B10] Dworak O Keilholz L Hoffmann A Pathological features of rectal cancer after preoperative radiochemotherapy Int J Colorectal Dis 1997 12 19 23 10.1007/s003840050072 9112145

[B11] Locker GY Hamilton S Harris J Jessup JM Kemeny N Macdonald JS Somerfield MR Hayes DF Bast RC Jr ASCO 2006 update of recommendations for the use of tumor markers in gastrointestinal cancer J Clin Oncol 2006 24 5313 5327 10.1200/JCO.2006.08.2644 17060676

[B12] Ishihara S Watanabe T Kiyomatsu T Yasuda K Nagawa H Prognostic significance of response to preoperative radiotherapy, lymph node metastasis, and CEA level in patients undergoing total mesorectal excision of rectal cancer Int J Colorectal Dis 2010 25 1417 1425 10.1007/s00384-010-1051-1 20809426

[B13] Das P Skibber JM Rodriguez-Bigas MA Feig BW Chang GJ Wolff RA Eng C Krishnan S Janjan NA Crane CH Predictors of tumor response and downstaging in patients who receive preoperative chemoradiation for rectal cancer Cancer 2007 109 1750 1755 10.1002/cncr.22625 17387743

[B14] Park YA Sohn SK Seong J Baik SH Lee KY Kim NK Cho CW Serum CEA as a predictor for the response to preoperative chemoradiation in rectal cancer J Surg Oncol 2006 93 145 150 10.1002/jso.20320 16425302

[B15] Yoon SM Kim DY Kim TH Jung KH Chang HJ Koom WS Lim SB Choi HS Jeong SY Park JG Clinical parameters predicting pathologic tumor response after preoperative chemoradiotherapy for rectal cancer Int J Radiat Oncol Biol Phys 2007 69 1167 1172 10.1016/j.ijrobp.2007.04.047 17967307

[B16] Kim CW Yu CS Yang SS Kim KH Yoon YS Yoon SN Lim SB Kim JC Clinical significance of pre- to post-chemoradiotherapy s-CEA reduction ratio in rectal cancer patients treated with preoperative chemoradiotherapy and curative resection Ann Surg Oncol 2011 18 3271 3277 10.1245/s10434-011-1740-1 21537868

[B17] Kouri M Pyrhonen S Mecklin JP Jarvinen H Laasonen A Franssila K Nordling S The prognostic value of DNA-ploidy in colorectal carcinoma: a prospective study Br J Cancer 1990 62 976 981 10.1038/bjc.1990.420 2257229 PMC1971551

[B18] Della Vittoria Scarpati G Falcetta F Carlomagno C Ubezio P Marchini S De Stefano A Singh VK D'Incalci M De Placido S Pepe S A specific miRNA signature correlates with complete pathological response to neoadjuvant chemoradiotherapy in locally advanced rectal cancer Int J Radiat Oncol Biol Phys 2012 83 1113 1119 10.1016/j.ijrobp.2011.09.030 22172905

[B19] Perez RO Sao Juliao GP Habr-Gama A Kiss D Proscurshim I Campos FG Gama-Rodrigues JJ Cecconello I The role of carcinoembriogenic antigen in predicting response and survival to neoadjuvant chemoradiotherapy for distal rectal cancer Dis Colon Rectum 2009 52 1137 1143 10.1007/DCR.0b013e31819ef76b 19581858

[B20] Park JW Lim SB Kim DY Jung KH Hong YS Chang HJ Choi HS Jeong SY Carcinoembryonic antigen as a predictor of pathologic response and a prognostic factor in locally advanced rectal cancer patients treated with preoperative chemoradiotherapy and surgery Int J Radiat Oncol Biol Phys 2009 74 810 817 10.1016/j.ijrobp.2008.08.057 19101093

[B21] Bonnen M Crane C Vauthey JN Skibber J Delclos ME Rodriguez-Bigas M Hoff PM Lin E Eng C Wong A Janjan NA Feig BW Long-term results using local excision after preoperative chemoradiation among selected T3 rectal cancer patients Int J Radiat Oncol Biol Phys 2004 60 1098 1105 10.1016/j.ijrobp.2004.04.062 15519780

